# Silibinin, a potential fasting mimetic, inhibits hepatocellular carcinoma by triggering extrinsic apoptosis

**DOI:** 10.1002/mco2.457

**Published:** 2024-01-11

**Authors:** Biying Xiao, Yanyu Jiang, Shuying Yuan, Lili Cai, Tong Xu, Lijun Jia

**Affiliations:** ^1^ Cancer Institute Longhua Hospital Shanghai University of Traditional Chinese Medicine Shanghai China; ^2^ Departmnent of Oncology Affiliated Hospital of Jiangnan University Wuxi China

**Keywords:** AMPK, apoptosis, fasting mimetic, hepatocellular carcinoma, silibinin

## Abstract

Fasting, without inducing malnutrition, has been shown to have various beneficial effects, including the inhibition of tumor initiation and progression. However, prolonged fasting poses challenges for many cancer patients, particularly those in intermediate and terminal stages. Thus, there is an urgent need for the development of fasting mimetics which harness the protective effects of fasting but more suitable for patients. In this study, we first highlighted the pivotal role of silibinin in AMP‐activated protein kinase (AMPK) pathway and may serve, as a potential fasting mimetic via screening hepatoprotective drugs. Further metabolic analysis showed that silibinin inhibited the adenosine triphosphate (ATP) levels, glucose uptake and diminished glycolysis process, which further confirmed that silibinin served as a fasting mimetic. In addition, fasting synergized with silibinin, or used independently, to suppress the growth of hepatocellular carcinoma (HCC) in vivo. Mechanistically, silibinin upregulated death receptor 5 (DR5) through AMPK activation, and thus promoting extrinsic apoptosis and inhibiting HCC growth both in vitro and in vivo. Inhibition of AMPK using small interfering RNA (siRNA) or compound C, an AMPK inhibitor, significantly attenuated the upregulation of DR5 and the apoptotic response induced by silibinin. These findings suggest that silibinin holds promise as a fasting mimetic and may serve as an adjuvant drug for HCC treatment.

## Graphical abstract

This study highlights the hepatoprotective drug silibinin triggers AMPK pathway, and inhibits intracellular ATP levels and glycolysis, which served as a fasting mimetic to induce extrinsic apoptosis and inhibit HCC growth. Silibinin achieves this effect partially by activating the AMPK–DR5 pathway. These findings suggest silibinin holds promise as a therapeutic agent to improve outcomes for HCC patients.

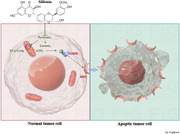

## 1 INTRODUCTION

Primary liver cancer, identified as the seventh most common cancer worldwide and the third deadliest malignancy, poses a significant health risk.^1^, ^2^, ^3^ HCC stands out as the most common histological subtype. Although surgical resections are the primary clinical approach, they are only suitable for patients diagnosed with early‐stage HCC. Molecular targeted therapy and immunotherapy, such as sorafenib, lenvatinib, nivolumab, have shown encouraging results in advanced‐stage HCC.^4^, ^5^ However, the prognosis for HCC remains poor, with a lower 5‐year overall survival rate.^2^, ^3^, ^6^ Thus, there is an urgent need for new interventions for liver cancer patients.

Fasting, without inducing malnutrition, has been shown to have various beneficial effects, including extension of lifespan,^7^, ^8^, ^9^ suppression of oxidative damage,^10^ inflammation,^11^, ^12^, ^13^, ^14^ and cancer progression.^15^, ^16^, ^17^ In 1994, Grasl‐Kraupp et al.^18^ reported that calorie restriction eliminated preneoplastic cells, as best supported by an 85% reduction in the number and volume of preneoplastic liver foci after fasting for 3 months. And then fasting has been emerged as an effective dietary intervention with the potential for the prevention or treatment of multiple tumors.^15^, ^19^, ^20^ For example, in 2009 and 2013, respectively, Kalaany et al.^21^ and Curry et al.^17^ reported that tumors without phosphoinositide 3‐kinase activation were sensitive to dietary restriction, whereas mutated in multiple advanced cancers 1‐null or ectonucleoside triphosphate diphosphohydrolase 5‐activated cancer cell lines were resistant to dietary restriction. In 2016, Di Biase et al.^12^ and Pietrocola et al.^22^ reported that fasting improved anti‐cancer immuno‐surveillance. Additionally, numerous studies have shown that fasting, either alone or in conjunction with chemotherapy, hinders the growth of various tumors.^23^, ^24^, ^25^, ^26^, ^27^, ^28^ Furthermore, fasting induces differential stress resistance, thereby protecting normal cells without extending the same protection to tumor cells.^10^ However, prolonged fasting poses challenges for many cancer patients, particularly those in intermediate and terminal stages. Alternative approaches such as calorie restriction mimetics (CRM) or fasting‐mimicking diets (FMD) have emerged to harness the protective effects of fasting.^29^, ^30^ CRM or FMD can achieve similar protective effects to fasting by reducing glucose, inducing autophagy, activating AMPK or reprogramming metabolic characteristics by inhibiting glycolysis process.^30^, ^31^, ^32^, ^33^

AMPK, an energy sensor, plays a crucial role in maintaining energy homeostasis during glucose starvation.^34^ In general, AMPK detects the increased adenosine monophophate (AMP)/ATP or adenosine diphosphate (ADP)/ATP ratio and is phosphorylated by upstream kinases.^30^, ^31^, ^32^, ^33^, ^35^ Phosphorylated AMPK (p‐AMPK) subsequently phosphorylated multiple targets, such as unc‐51 like autophagy activating kinase 1 (ULK1), to inhibit anabolism and promote catabolism.^32^, ^35^ This process minimizes ATP consumption while stimulating ATP production, thus ensuring energy homeostasis.^31^ In 2022, the research group led by Sheng‐Cai Lin reported that aldometanib, an aldolase inhibitor, activates AMPK, simulating a cellular state of glucose starvation, and AMPK might serve as a potential therapeutic target in humans.^36^ Metformin, a primary drug for treating type 2 diabetes, is known for its AMPK activation and also recognized as a fasting mimetic.^37^, ^38^ Aspirin, the inhibitor of enzyme cyclooxygenase and defined as another caloric‐restriction mimetic, could inhibit non‐small cell lung cancer cells and liver cancer cells growth by activating AMPK pathway.^15^, ^39^, ^40^ Meanwhile, traditional Chinese medicine extracted compounds (such as: 20(S)‐protopanaxadiol^41^, ^42^ and resveratrol^43^, ^44^) are detected as fasting mimetics to activate AMPK pathway.^45^ Given AMPK's involvement in modulating metabolic dysregulation, mitochondrial dynamics and functions—hallmarks of cancer, AMPK presents as a promising target for cancer therapies. Our previous clinical trial showed that fasting inhibited glycolysis with a decrease in glucose, fructose, pyruvate and lactate levels.^46^ Aberrant metabolism has been considered a hallmark of cancer cells,^47^, ^48^ and cancer cells derive most of their energy from glycolysis rather than mitochondrial oxidative phosphorylation as their primary energy resource.^49^ Fasting inhibits glycolysis, lactate production and reprogram metabolic derangements to inhibit growth of cancer cells,^50^, ^51^ which could also be the characterizations and mechanisms of fasting mimetics.

In the present study, we highlighted the pivotal role of hepatoprotective drug silibinin in AMPK pathway, which served as a potential fasting mimetic. Further metabolic analysis showed that silibinin decreased the intracellular ATP level, glucose uptake level and diminished glycolysis process, which further confirmed silibinin was a fasting mimetic. Meanwhile, silibinin synergized with fasting, or used independently, to suppress the growth of HCC of fasting via activating AMPK pathway and inhibiting HCC growth both in vitro and in vivo. Additionally, silibinin upregulated DR5 through AMPK activation, and promoted extrinsic apoptosis. Inhibition of AMPK significantly attenuated the upregulation of DR5 and the apoptotic response induced by silibinin. These findings suggests that silibinin holds promise as a fasting mimetic and may serve as an adjuvant drug for HCC treatment.

## 2 RESULTS

### 2.1 Fasting inhibits HCC progression and activates AMPK pathway

**FIGURE 1:**
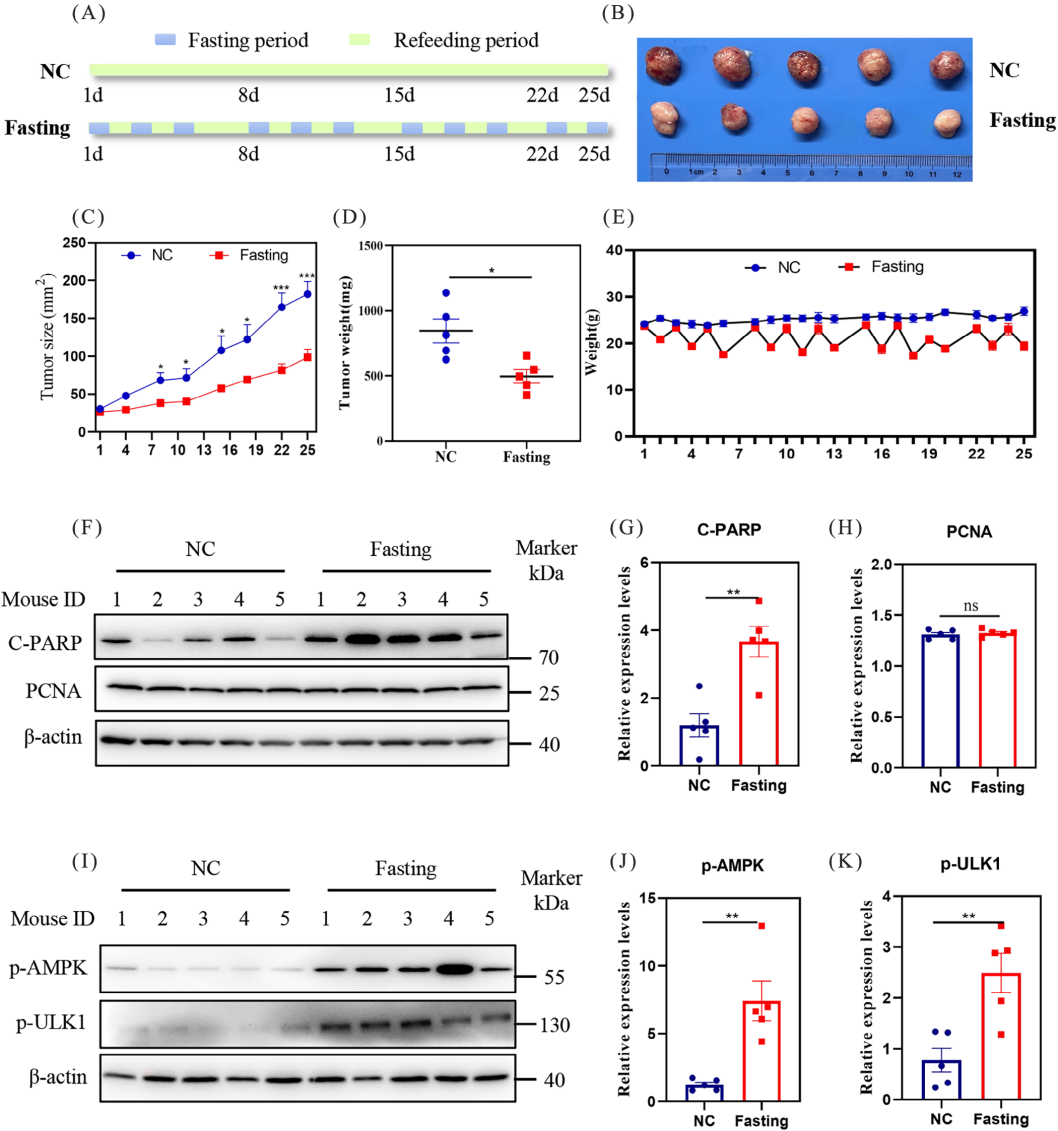
Fasting inhibits HCC progression and activates AMPK pathway in vivo. (A) Schematic representation of fasting regimen in balb/c nude mice xenograft tumors model. (B) Mice subcutaneous tumor tissues were harvested and photographed. (C) Mice subcutaneous tumor size. (D) Mice tumor weight. (E) Mice body weight. (F–I) Tissue proteins were extracted and detected by western blot (G and H, J and K) The band density of tissue proteins.

To assess the potential inhibitory impact of fasting on HCC progression in vivo, we utilized HCC‐LM3 cell lines to establish a xenograft mouse model (Figure 1A). Our findings revealed that fasting treatment significantly abated the tumor growth which was evidenced by tumor size (Figures 1B and C) and tumor weight (Figure 1D). During the fasting period, the mice experienced a 20% decrease in body weight, which was subsequently recovered to the baseline level after one day of refeeding (Figure 1E). We then assessed whether fasting induced cell death or inhibited cell proliferation. As shown, the protein levels of cleaved PARP (C‐PARP) were significantly increased, while the proliferation marker proliferating cell nuclear antigen (PCNA) showed no significant changes (Figures 1F–H). These results illustrate that fasting may induce apoptosis to limit HCC growth.

Considering the pivotal role of AMPK as an energy sensor in sustaining energy homeostasis and fine‐tuning multiple biological processes during fasting,^31^, ^33^ we examined the phosphorylation state of AMPK. The results showed that fasting significantly increased the protein levels of p‐AMPK and its classical substrates phosphorylated ULK1 (p‐ULK1) (Figures 1I–K), indicating the activation of the AMPK pathway by fasting.

### 2.2 Discovery of optimal AMPK activator silibinin

**FIGURE 2:**
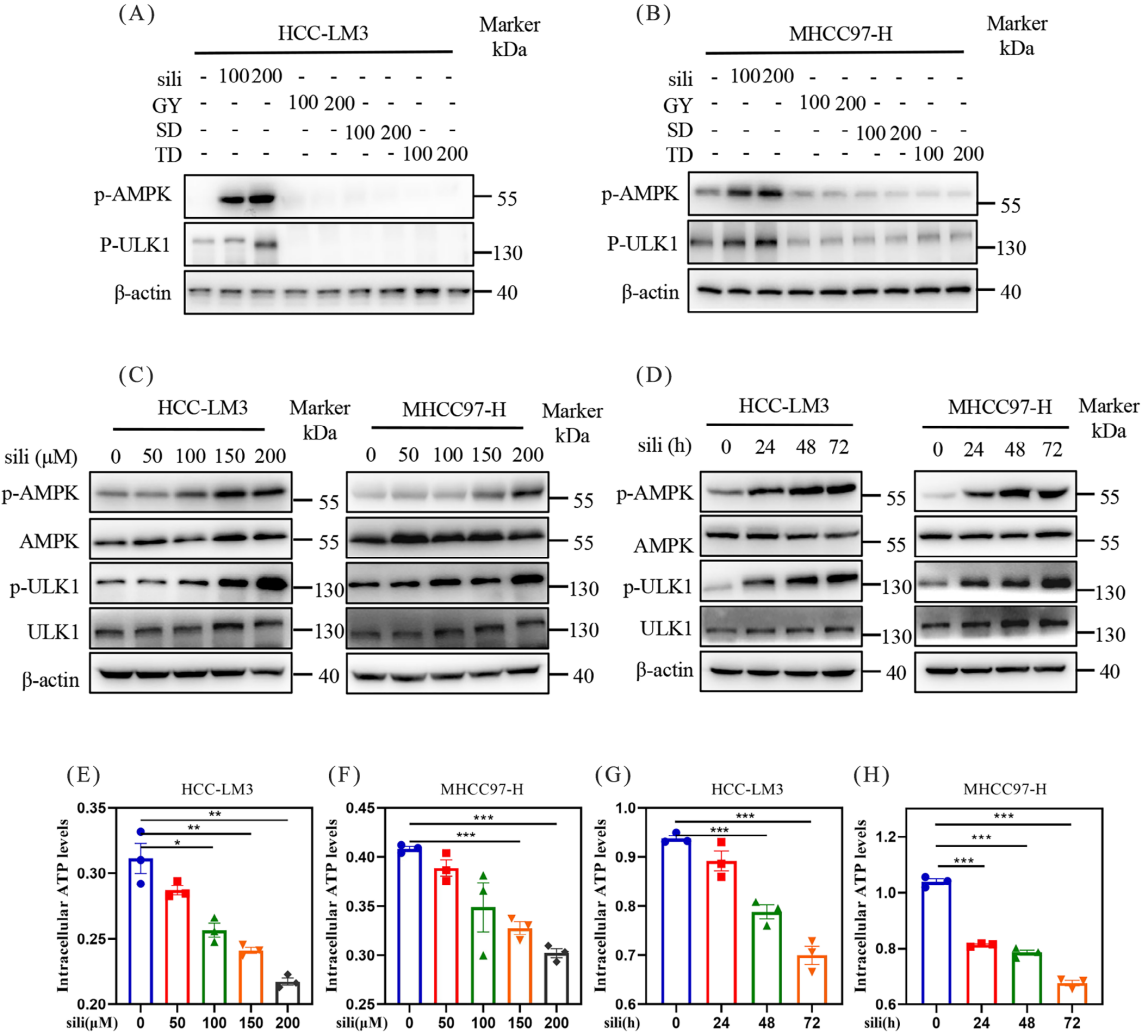
Silibinin, a potential fasting mimic, activates AMPK pathway. (A and B) HCC‐LM3 and MHCC97‐H cells were treated with silibinin (μM), GY(μM), SD (μM), and TD (μM) as described above, and the protein levels were detected by western blot. (C and D) HCC‐LM3 and MHCC97‐H cells were incubated with silibinin at indicated concentration (C) or time (D), the AMPK pathway‐related protein levels were tested by western blot. (E–H) HCC‐LM3 and MHCC97‐H cells were incubated with silibinin at indicated concentration (E and F) or time (G and H), and the intracellular ATP levels were evaluated.

To identify potential fasting mimetics, we treated HCC‐LM3 and MHCC97‐H cells with some hepatoprotective drugs whose functions resembled the protective effects of fasting: diammonium glycyrrhizinate (GY),^52^, ^53^ silibinin,^54^, ^55^ schisandrin (SD),^56^, ^57^ and tiopronin (TD).^58^ The findings illustrated that silibinin significantly upregulated the protein levels of p‐AMPK and p‐ULK1, whereas other hepatoprotective drugs, such as GY, SD, and DG, did not upregulated p‐AMPK and p‐ULK1 (Figures 2A and B). Silibinin, a natural polyphenolic compound extracted from the seeds of herb *milk thistle*, which was widely used as hepatoprotective drugs in the treatment of liver diseases such as fibrosis,^59^ cirrhosis,^60^ hepatitis,^61^ alcoholic liver disease,^62^ non‐alcoholic fatty liver disease (NAFLD),^63^ and toxin exposure.^64^ We next treated HCC‐LM3 and MHCC97‐H cells with silibinin using a series of doses at different time. The results demonstrated that silibinin upregulated p‐AMPK in both dose‐ and time‐dependent manner in HCC‐LM3 and MHCC97‐H cell lines (Figures 2C and D), indicating that silibinin was an AMPK activator. To further ascertain whether silibinin could act as an AMPK inhibitor, we evaluated the intracellular ATP levels in HCC‐LM3 and MHCC97‐H cells. The ATP assays revealed that silibinin treatment significantly diminished intracellular ATP levels in both MHCC97‐H and HCC‐LM3 cell lines in a time‐ and dose‐dependent manner (Figures 2E–H). Collectively, these findings indicate that silibinin activates AMPK and decreases the intracellular ATP levels in HCC cells, two features of fasting.

### 2.3 Metabolic profiling demonstrates silibinin as a fasting mimetic

**FIGURE 3:**
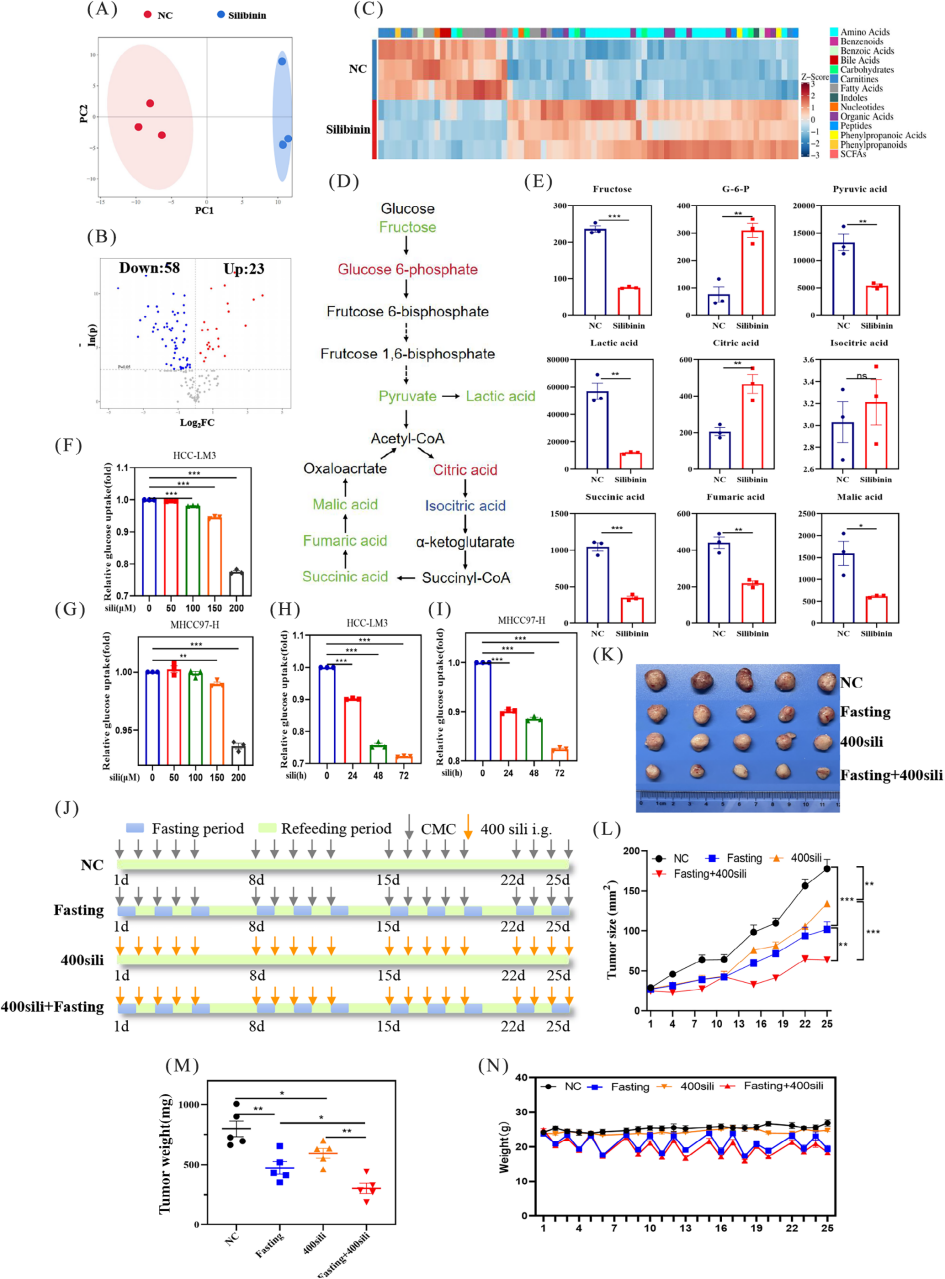
Silibinin served as a potential fasting mimetic suppressing the growth of HCC. (A) Principal‐component analysis of silibinin (200 μM, 48 h) or DMSO treated MHCC97‐H cells. (B) Volcano plot analysis. (C) Heatmap analysis of significantly altered metabolites (*p* < 0.05). (D and E) Glucose metabolism was differentially expressed after silibinin treatment, including fructose, G‐6‐P, pyruvate acid, lactic acid, citric acid, succinic acid, fumaric acid, and malic acid. Green denotes downregulated; red denotes upregulated; and blue denotes not changed. Black denotes not detected. (F–I) HCC‐LM3 and MHCC97‐H cells were incubated with silibinin at indicated concentration (F and G) or time (H and I), and the level of glucose uptake were evaluated. (J) Schematic representation of fasting and silibinin regimen in balb/c nude mice xenograft tumors model. (K) Mice subcutaneous tumor tissues were harvested and photographed. (L) Mice subcutaneous tumor size. (M) Mice tumor weight. (N) Mice body weight during the treatment.

To further ascertain that silibinin is a fasting mimetic, we next examined the changes in fasting‐related metabolic pathways after silibinin treatment. Initially, we conducted metabolomic profiling and the results demonstrated that numerous metabolites were altered after silibinin treatment, as elucidated by principal component analysis (PCA), volcano plots, and heatmap analysis (Figures 3A–C). This analysis identified 81 differentially expressed metabolites, with 23 being upregulated and 58 downregulated (Figure 3B). We then assessed the metabolite levels within the glycolysis and tricarboxylic acid (TCA) cycle following silibinin treatment. As depicted in Figure 3D, significant alterations were observed in the glycolysis and TCA cycle pathways. Specifically, three metabolites in glycolysis (fructose, pyruvate, and lactic acid) and three in the TCA cycle (succinic acid, fumaric acid, and malic acid) were decreased, while glucose‐6‐phosphate(G‐6‐P) in glycolysis and citric acid in the TCA cycle were increased (Figures 3D and E). Moreover, we performed glucose consumption assay, and the results showed that silibinin treatment significantly curtailed glucose uptake levels in MHCC97‐H and HCC‐LM3 cells in both time‐ and dose‐dependent manner (Figures 3F–I). These findings suggest that glycolysis and the TCA cycle were inhibited post‐silibinin treatment, further bolstering the proposition that silibinin could serve as a potential fasting mimetic.

### 2.4 Silibinin synergizes with fasting, or used independently, to suppress the growth of HCC

Considering the activation of AMPK pathway, reduction of intracellular ATP levels, decreasing glucose uptake ability and reprogramming metabolism pathway by both silibinin and fasting, we hypothesized that the combination of silibinin and fasting could synergistically inhibit the growth of HCC cells. In vivo, silibinin (400 mg/kg/day) alone or combined with fasting (Figure 3J) significantly suppressed tumor growth, and as evidenced by the analysis of tumor size (Figures 3K and L) and tumor weight (Figure 3M). Moreover, the body weight of the fasting group and the combined treatment group decreased during the fasting period but returned to baseline after one day of refeeding (Figure 3N). No significant reduced in body weight were observed within the silibinin group (Figure 3N). These findings indicated that silibinin holds potential as a fasting mimetic to inhibit tumor growth, whether used independently or in conjunction with fasting.

### 2.5 Silibinin inhibits the growth of HCC cells via inducing apoptosis

**FIGURE 4:**
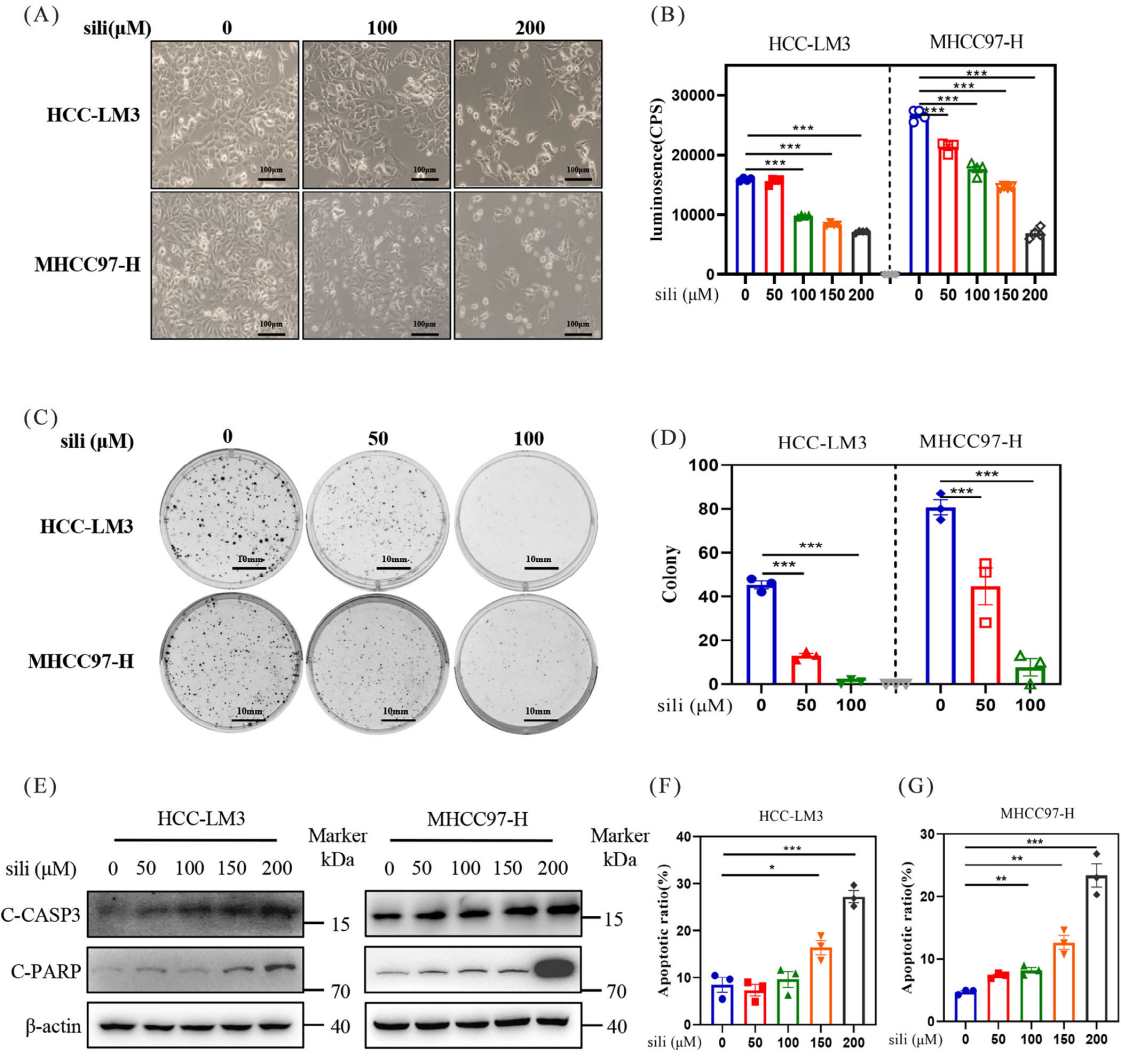
Silibinin inhibits the growth of HCC cells via inducing apoptosis. (A) Representative image of human HCC‐LM3 and MHCC97‐H cells which were treated with indicated concentrations of silibinin for 48 h, scale bar: 100 μm. (B) Cell viability assay of silibinin in HCC‐LM3 and MHCC97‐H cell lines were detected by ATPlite. (C) Representative images of colony formation by silibinin treated HCC‐LM3 and MHCC97‐H cell lines, scale bar: 10 mm. (D) The statistical graph of the relative number of colony formation. (E) The protein levels of C‐CASP3 and C‐PARP in HCC‐LM3 and MHCC97‐H cells were detected by western blot. (F and G) HCC‐LM3 and MHCC97‐H cells were treated with indicated concentrations of silibinin for 72 h, and then analyzed the proportion of apoptotic cells with FCAS, and the statistical graph was made.

To clarify the underlying mechanisms of silibinin in suppressing the growth of HCC cells, we investigated the cellular response of silibinin. First, we examined the impact of silibinin on the growth of two distinct HCC cell lines in vitro. The experimental results indicated a dose‐dependent suppression of cell proliferation (Figures 4A and B) and colony formation (Figures 4C and D) by silibinin in HCC‐LM3 and MHCC97‐H cell lines. Subsequently, we assessed the level of apoptosis following silibinin treatment in HCC‐LM3 and MHCC97‐H cell lines. Silibinin treatment significantly upregulated the classical apoptosis markers cleaved caspase 3 (C‐CASP3) and C‐PARP in HCC (Figure 4E). Moreover, the population of Annexin V‐FITC‐positive cells significantly increased after 72 h of silibinin treatment (Figures 4F and G). These results demonstrated that silibinin significantly inhibit the growth of HCC cells via inducing apoptosis.

### 2.6 Silibinin‐triggered apoptosis is mediated by AMPK in HCC cell lines

**FIGURE 5:**
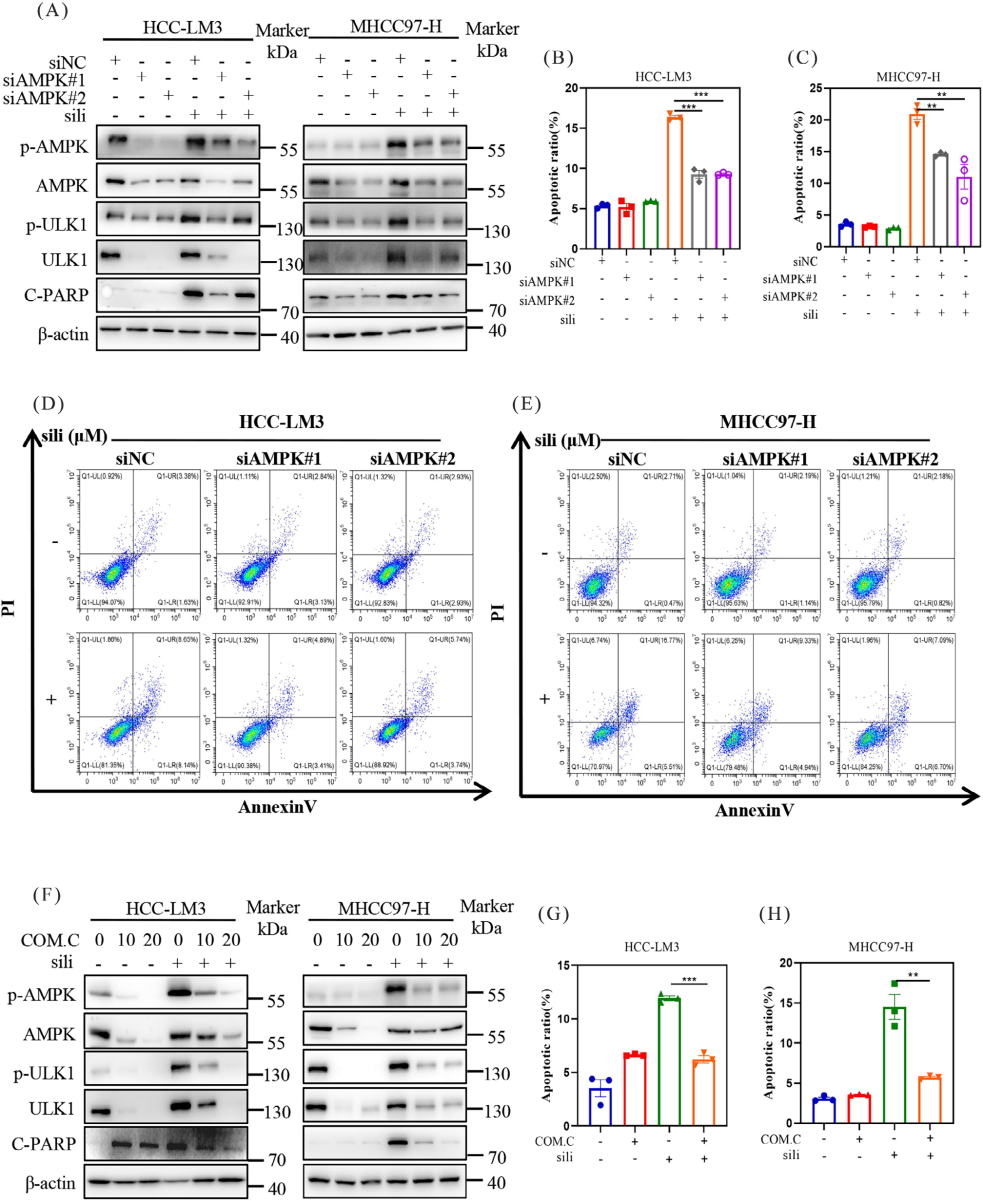
Silibinin induced apoptosis is mediated by AMPK in HCC cell lines. (A) HCC‐LM3 and MHCC97‐H cells were transfected with control or two siRNA sequences of AMPK, then treated with silibinin (200 μM) for 48 h. AMPK‐related pathway and C‐PARP protein levels were detected by western blot. (B–E) HCC‐LM3 (B and D) and MHCC97‐H (C and E) cells were knockdown AMPK and then treated with silibinin to detect apoptosis induction, and the statistical graph was made. (F) HCC‐LM3 and MHCC97‐H cells were treated with Compound C (10 μM) and silibinin (200 μM) to determine the AMPK‐related pathway and C‐PARP protein levels by western blot. (G and H) Apoptosis induction in silibinin treated with Compound C (10 μM) in HCC‐LM3 and MHCC97‐H cell lines.

To explore the involvement of AMPK in apoptosis triggered by silibinin treatment, we utilized siRNAs to suppress AMPK expression and assessed the impact on silibinin‐induced apoptosis in HCC cell lines. We found that downregulation of AMPK decreased the protein level of C‐PARP after silibinin treatment both in HCC‐LM3 and MHCC97‐H cell lines (Figure 5A). These results demostrated that AMPK mediated silibinin‐induced apoptosis. These findings were further supported by flow cytometry analysis, which showed that knock down of AMPK using siRNA inhibited silibinin‐induced apoptosis, as evidenced by a significant decrease in Annexin V‐FITC‐positive cells (Figures 5B–E).

Next, we utilized Compound C to inhibit the AMPK pathway and found that it reversed the silibinin‐induced apoptosis in a dose‐dependent manner in HCC‐LM3 and MHCC97‐H cell lines (Figure 5F). Consistently, combined silibinin with Compound C resulted in a significant reduction in Annexin V‐FITC‐positive cells (Figures 5G and H). These results provide further evidence that the apoptotic effect of silibinin in HCC cells is mediated through AMPK.

### 2.7 Silibinin triggers extrinsic apoptosis in HCC cell lines

**FIGURE 6:**
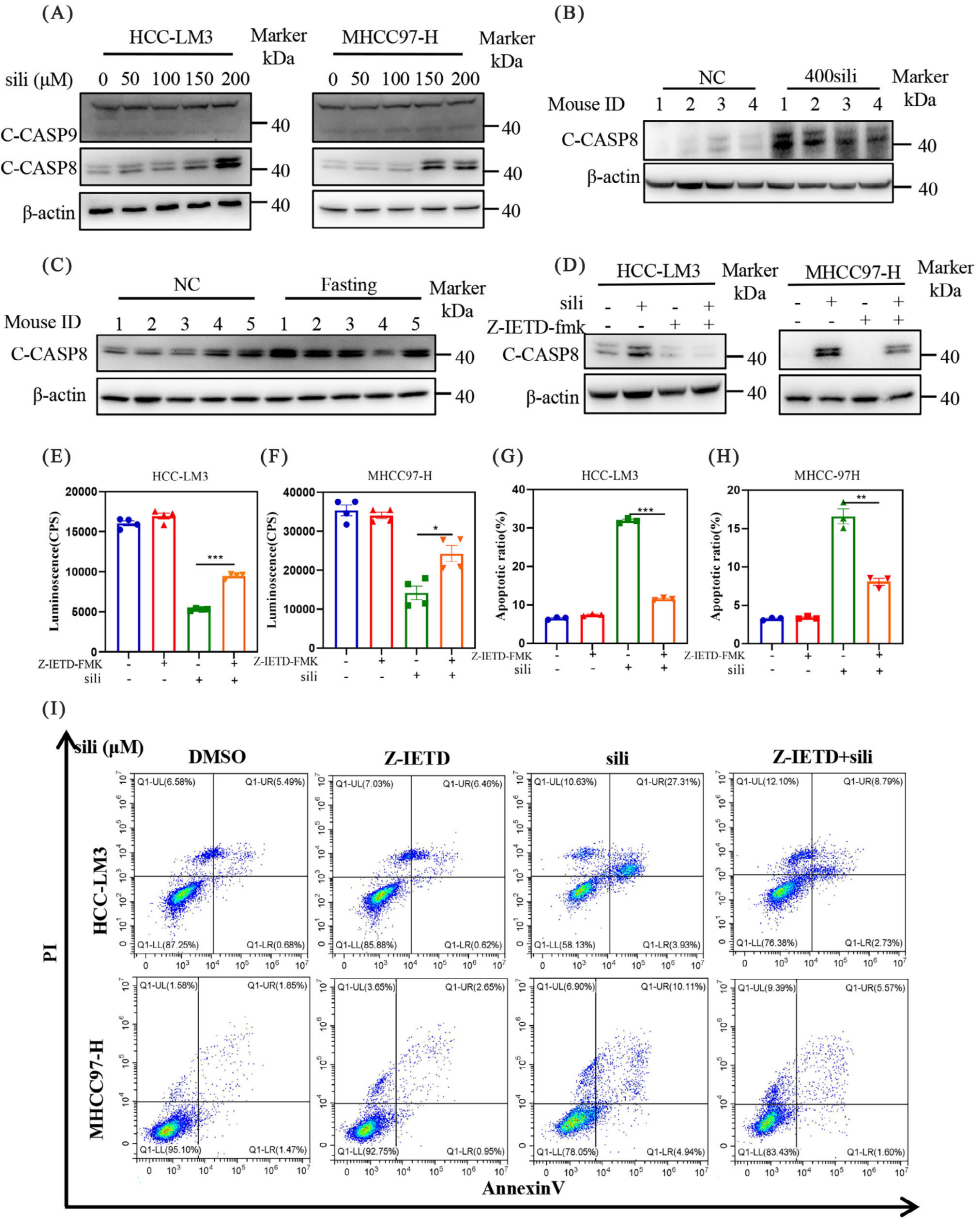
Silibinin triggers extrinsic apoptosis in HCC cell lines. (A) After silibinin treatment, the protein levels of C‐CASP9 and C‐CASP8 were detected in HCC‐LM3 and MHCC97‐H cells. (B and C) Mice tumor tissue was extracted and the protein levels of C‐CASP8 were detected separately. (D) After silibinin with or without Z‐IETD (20 μM) treatment the C‐CASP8 protein levels were detected, and cell viability (E and F) were detected. (G–I) Apoptosis induction was quantified by Annexin V‐FITC/PI double‐staining analysis, and the statistical graph was made.

To elucidate the molecular mechanisms, we assessed the expression of cleaved caspase 8 (C‐CASP8), a classical marker of extrinsic apoptosis, and cleaved caspase 9 (C‐CASP9), a classical marker of intrinsic apoptosis. Our findings revealed that silibinin treatment resulted in the accumulation of cleaved caspase 8 in a dose‐dependent manner, while it had no effect on cleaved caspase 9 levels in both HCC‐LM3 and MHCC97‐H cell lines (Figure 6A). This observation was further supported by in vivo experiments, which silibinin and fasting induced the accumulation of C‐CASP8 (Figures 6B and C). These results suggest that both fasting and silibinin might activate extrinsic apoptosis.

To further validate the underlying mechanisms of extrinsic apoptosis by silibinin, we incubated specific extrinsic apoptosis inhibitor Z‐IETD‐FMK (Z‐IETD) with silibinin in HCC‐LM3 and MHCC97‐H cell lines. As shown in Figure 6D, the addition of Z‐IETD led to a downregulation of silibinin‐induced C‐CASP8 expression. Consistently, the results from cell viability assays (Figures 6E and F) and flow cytometry analysis of Annexin V‐FITC‐positive cells (Figures 6G–I) demonstrated that Z‐IETD significantly rescued the silibinin‐induced extrinsic apoptosis. These findings collectively provide strong evidence that silibinin triggers extrinsic apoptosis, thereby inhibiting tumor growth.

### 2.8 Silibinin upregulates DR5 to induce extrinsic apoptosis in HCC cell lines

**FIGURE 7:**
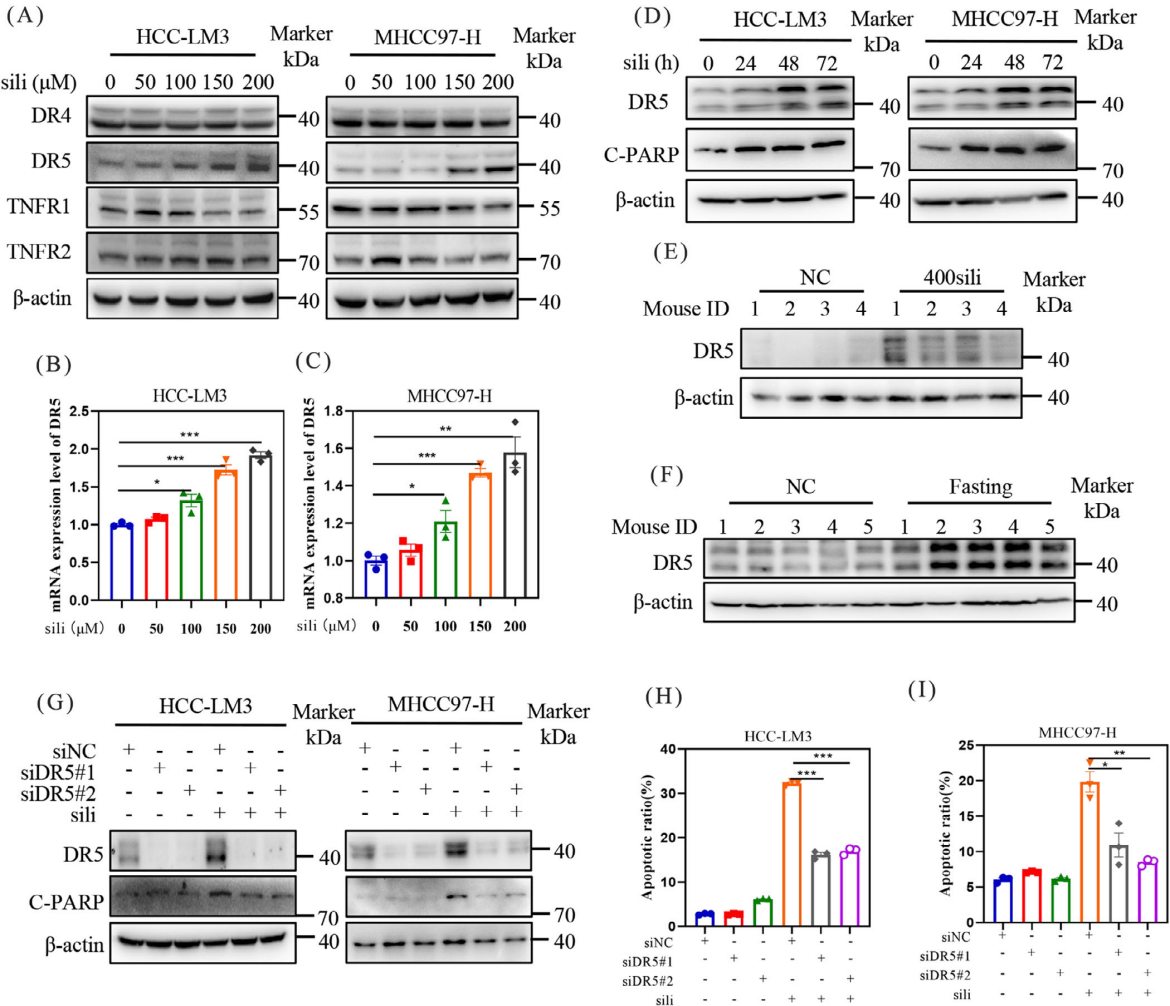
Silibinin triggers extrinsic apoptosis in DR5‐dependent manner. (A) HCC‐LM3 and MHCC97‐H cells were treated with indicted concentrations, and proteins were detected by western blot. (B and C) The mRNA levels of DR5 were detected by q‐PCR. (D) The proteins levels of DR5 and C‐PARP was quantified by western blot. (E and F) Mice tumor tissue was extracted and detected DR5 expression level by western blot separately. (G) DR5 and C‐PARP protein levels in silibinin treated DR5‐knockdown HCC‐LM3 and MHCC97‐H cell lines. (H and I) Apoptosis induction was quantified by Annexin V‐FITC/PI double‐staining analysis.

**FIGURE 8:**
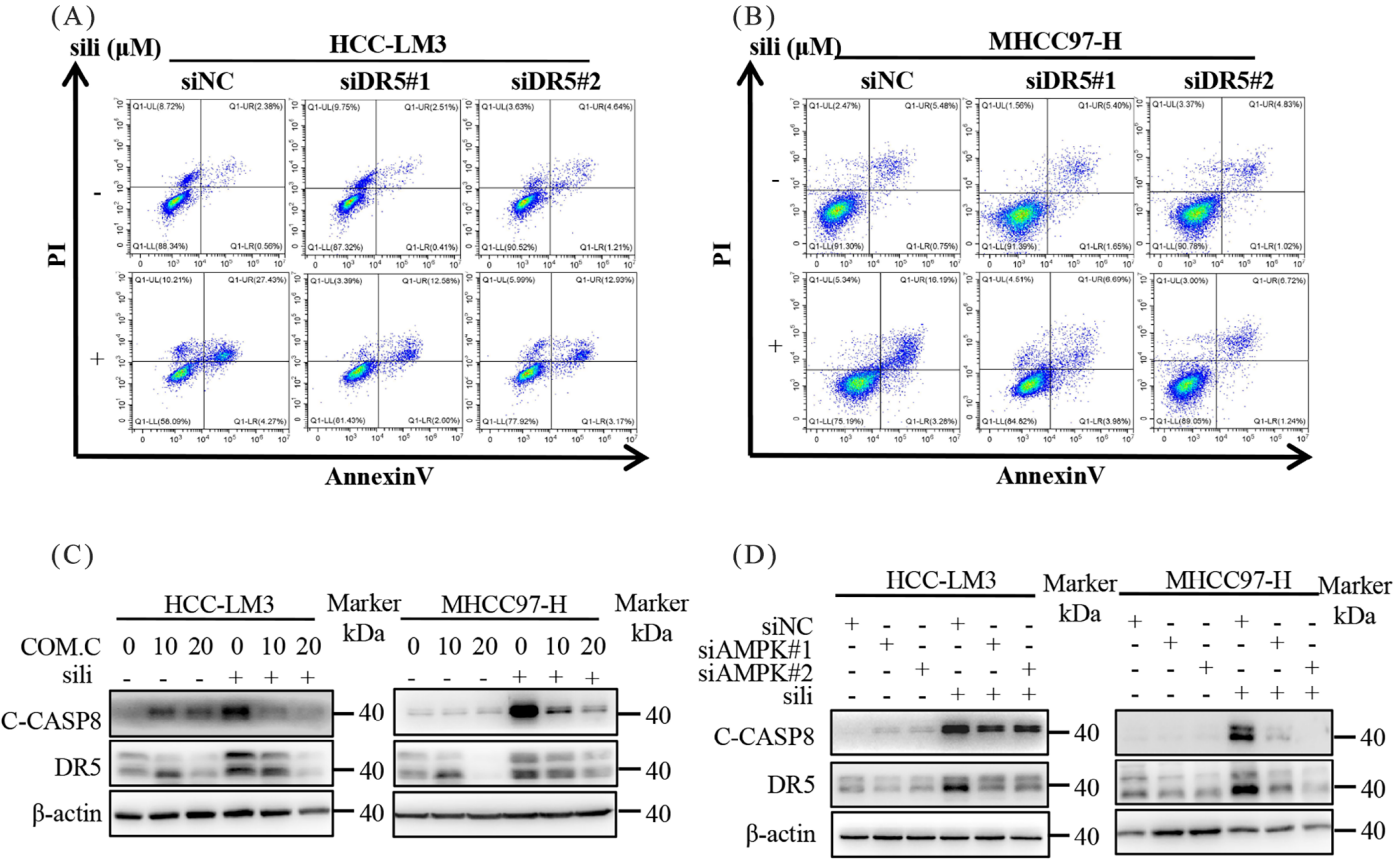
Silibinin triggers extrinsic apoptosis via AMPK–DR5 axis. (A and B) Apoptosis induction in silibinin treated DR5‐knockdown HCC‐LM3 and MHCC97‐H cell lines. (C) DR5 and C‐CASP8 protein levels of silibinin and Compound C treatment in HCC‐LM3 and MHCC97‐H cell lines. (D) DR5 and C‐PARP protein levels in silibinin treated AMPK‐knockdown HCC‐LM3 and MHCC97‐H cell lines.

To investigate the mechanisms underlying silibinin‐induced extrinsic apoptosis, we examined the expression of death receptors and ligands, including death receptor 4 (DR4), DR5, tumor necrosis factor receptor‐1 (TNFR1), and tumor necrosis factor receptor‐2 (TNFR2) (Figure 7A). Among these proteins, silibinin dose‐dependently stimulated the expression of DR5 (Figures 7A–C). Furthermore, a time‐dependently increase in DR5 accumulation was observed in both HCC‐LM3 and MHCC97‐H cell lines after silibinin treatment (Figure 7D). Consistent with these findings, analysis of tumor samples from mice confirmed that silibinin and fasting treatment led to the accumulation of DR5 compared with the control group (Figures 7E and F). Based on these results, we hypothesize that the induction of extrinsic apoptosis by silibinin is dependent on DR5. To validate this hypothesis, we downregulated DR5 using siRNA in silibinin‐treated HCC‐LM3 and MHCC97‐H cells. The results demonstrated that downregulation of DR5 reduced the levels of C‐PARP (Figure 7G) and partially reversed the silibinin‐induced apoptosis in HCC‐LM3 and MHCC97‐H cells (Figures 7H, I and 8A, B). These results indicates that silibinin induced DR5‐mediated extrinsic apoptosis.

Finally, we investigated the role of AMPK in silibinin‐induced extrinsic apoptosis. Silibinin treatment significantly increased DR5 expression, and this effect was attenuated by Compound C, an inhibitor of p‐AMPK, as well as by AMPK knockdown (Figures 8C and D). Similarly, inhibition of AMPK activation through pharmacological or genetic methods significantly attenuated the increase in C‐CAPS8 induced by silibinin (Figures 8C and D). Collectively, these results indicate that silibinin triggers extrinsic apoptosis through the AMPK–DR5 axis.

## 3 DISCUSSION

Fasting without malnutrition has been reported to exhibit anti‐cancer effects^12^, ^17^, ^65^ and has the potential to reduce risk factors associated with cancer, such as insulin‐like growth factor 1(IGF‐1),^46^ and glucose level.^66^ Interestingly, our previous clinical studies have shown that 5‐day water‐only fasting is safe and has the potential to decrease some cancer‐related risk factors (for example: glucose^66^). However, the anti‐cancer effects of fasting in tumors are still a subject of dispute. In the present study, we demonstrated that fasting inhibited HCC tumor growth in vivo without causing obvious adverse effects.

Nowadays, some clinical studies have been assessed the potential of fasting in cancer. For instance, Marinac et al.^67^ reported that fasting for less than 13 h per night was associated with a 36% increased risk of breast cancer recurrence, compared with fasting for 13 h or more per night. Bauersfeld et al.^68^ reported that short‐term fasting during chemotherapy was well‐tolerated and seemed to enhance quality of life and alleviate fatigue during chemotherapy. In the clinical trial website, two fasting‐related clinical trials have been approved, including NCT01954836 for gynecological cancer, and NCT00936364 for combination with cisplatin and gemcitabine. However, the clinical studies assessing the potential of fasting in cancer are still scarce. The reason may be attributed to that prolonged fasting poses challenges for many cancer patients, particularly those in intermediate and terminal stages. In addition, some clinical trials revealed that fasting (e.g., Ramadan fasting) is not suitable for patients who have chronic liver disease (e.g., non‐alcoholic steatohepatitis or cirrhosis).^69^, ^70^ Therefore, alternative approaches such as fasting mimetics or FMD have emerged to harness the benefits of fasting, and been advanced into clinical trials.^71^ In the clinical trial website, more than two FMD‐related clinical trials have been approved, including NCT04292041 for prostate cancer, NCT03700437 for non‐small cell lung cancer, NCT05384444 for colorectal cancer, NCT02126449 for human epidermal growth factor receptor 2‐negative breast cancer, NCT04248998 for triple‐negative breast cancer, and NCT05921149 for ovarian cancer. So many approved clinical trials further intensified the feasibility and acceptable of FMD other than fasting.

Silibinin, also known as silybin, is a natural polyphenolic compound extracted from the seeds of the herb *milk thistle*. Over the last 50 years, silibinin has been widely used in the treatment of liver diseases such as fibrosis,^59^ cirrhosis,^60^ hepatitis,^61^ alcoholic liver disease,^62^ NAFLD,^63^ and toxin exposure.^64^ Research conducted by Barzaghi et al.^72^ and Weyhenmeyer et al.^73^ employed the high‐performance liquid chromatography method to ascertain the clinical blood concentration of silibinin. The results revealed that the area under the curve from 0 to 12 h (AUC_0–12 h_) for silibinin (360 mg) was 257 ± 66 ng/mL h and the AUC_0–26 h_ of silibinin (245.3 mg) was 6807 ng/mL h. Moreover, a daily dosage of 2.1 g silibinin for 48 weeks showed a reduction in liver fibrosis in NAFLD patients (NCT02006498).^74^ Additionally, a daily dosage of 13 g of silibinin has been deemed safe for men diagnosed with prostate cancer (NCT00487721).^75^ According to the drug dose transformation between mice and human,^76^ the dosage of 13 g used in prostate cancer patients is equal to approximately 1 g in mice, exceeding the dosage (0.4 g/kg) we administered in mice. These data suggest that the dose of silibinin was suitable in the present study without dose‐related cytotoxicity. Recent studies have shown that silibinin inhibits tumor growth,^77^ tumor angiogenesis,^78^ epithelial‐to‐mesenchymal transition,^79^ and migration and invasion.^80^ Mechanistically, previous studies have reported that silibinin acts as an efficient inhibitor of heat shock protein 90 and signal transducer and activator of transcription 3 to inhibit tumor growth.^81^, ^82^ However, the molecular mechanisms underlying the induction of apoptosis in cancer cells by silibinin remain unclear. In the present study, we demonstrate that silibinin serves as a fasting mimetic to inhibit HCC growth alone, or the combination of fasting. In mechanism, silibinin acts as a cytotoxic agent, inducing extrinsic apoptosis through the AMPK–DR5 pathway.

Eukaryotes have developed a sophisticated system to sense low levels of cellular ATP through AMPK complex. Under low energy conditions, AMPK phosphorylates specific enzymes, such as ULK1, to increase ATP generation and decrease ATP consumption.^32^ Previous studies have shown that AMPK phosphorylates the proapoptotic caspase‐6 protein, inhibiting its activation and inducing hepatocyte apoptosis.^83^ In addition, AMPK could induce apoptosis by activating the AMPK–sirtuin 1 pathway or triggering reactive oxygen species‐associated apoptosis in various cancers.^54^ In the present study, we discovered that silibinin activates the AMPK pathway, leading to the upregulation of DR5 and the induction of extrinsic apoptosis. This represents a novel mechanism of AMPK‐related apoptosis. Previous studies have reported that AMPK restores krüppel‐like transcription factor 2 (KLF2), and knockdown of KLF2 reduces protein levels of DR5, suggesting that AMPK may regulate DR5 through KLF2.^84^

Fasting mimetics can achieve similar protective effects to fasting by activating AMPK or autophagy, or reducing glucose, or IGF‐1 as well as inducing metabolic reprogramming.^29^, ^85^, ^86^ Considering that AMPK serves as an energy sensor and holds a crucial role in glucose sensing and energy regulation, we first screened silibinin as an optimal AMPK activator. This function is consistently with four well‐known fasting mimetics, metformin,^29^, ^87^ aspirin,^39^, ^40^, ^88^ 20(S)‐protopanaxadiol,^41^, ^42^ and resveratrol.^43^, ^44^ To further demonstrate that silibinin is a fasting mimetic, we determined the intracellular ATP levels and glucose consumption. The results showed silibinin significantly inhibits these two fasting‐related hallmarks. Further metabolomic profiling illustrated silibinin inhibited glycolysis, a similar metabolic reprogramming characterization with our previous clinical trials.^46^ Activation of autophagy is a feature of fasting mimetics, thus we further analysis the relationship between silibinin and autophagy. Previous investigation elucidated that silibinin induced autophagy via enhancing AMP/ATP ratio, which transduced inhibitory signals to phosphatidylinositol‐4,5‐bisphosphate 3‐kinase catalytic subunit alpha‐protein kinase B–mammalian target of Rapamycin (mTOR) pathway in colorectal cancer cells.^89^ In addition, silibinin inhibits hypoxia‐inducible factor‐1*α* through the mTOR–ribosomal protein S6 kinase–4E‐binding protein‐1 pathway in human cancer cells to induce autophagy.^90^ In the present study, we found silibinin activate AMPK, which inhibit mTOR to activate autophagy in energy‐limited situations, indicating silibinin could induce autophagy. Combination with these features of fasting on reducing glucose levels and intracellular ATP levels, activating autophagy and AMPK pathway, inducing metabolic reprogramming by the suppression of glycolysis, we draw a conclusion that silibinin is a fasting mimetic.

In conclusion, our present study highlights the hepatoprotective drug silibinin triggers AMPK pathway, inhibits intracellular ATP levels and glycolysis, which served as a fasting mimetic to induce extrinsic apoptosis and inhibit HCC growth. Silibinin achieves this effect partially by activating the AMPK–DR5 pathway. These findings suggest silibinin holds promise as a fasting mimetic and therapeutic agent to improve outcomes for HCC patients.

## 4 MATERIALS AND METHODS

### 4.1 Cells

The human HCC cell lines, HCC‐LM3 (FH0096) and MHCC97‐H (FH0095), were purchased from FuHeng Cell Center, Shanghai, China.

### 4.2 Reagents

Silibinin, obtained from Sigma–Aldrich, was prepared for in vitro studies by dissolving it in dimethylsulfoxide and storing it at −20°C. For mice experiment, silibinin was dissolved in 0.5% sodium carboxymethyl cellulose (CMC) in sterile water. Compound C was procured from Selleck, and Z‐IETD‐FMK (Z‐IETD) was obtained from MCE (MedChemExpress).

### 4.3 Cell viability and clonogenic survival assay

Cells were cultured in triplicate in 96‐well plates at a density of 2500 cells per well and incubated with either dimethylsulfoxide or silibinin. Cell proliferation was evaluated using the Luminescence Assay Kit from PerkinElmer.

In the clonogenic assay, 500 cells were seeded in six‐well plates and treated with either dimethylsulfoxide or silibinin. After a 10‐day incubation period, cells were fixed and stained. Colonies, each containing more than 50 cells, were counted and photographed. This entire experimental process was independently replicated three times to ensure the reliability and accuracy of the results.

### 4.4 Apoptosis assay

Following 72 h of silibinin treatment, cells were harvested and washed (precooling PBS) three times. The cells were then exposed to Annexin V‐FITC/PI (ShareBio) and incubated for 30 min in a light‐protected setting. Subsequently, the flow cytometry equipment from Beckman Coulter was employed to quantify the apoptotic cell population.

### 4.5 Western blot analysis

The primary antibodies used in this study included: p‐AMPK (50081), AMPK (2532), P‐ULK1(5869), ULK1(8054), c‐PARP(94885), cleaved caspase 3(9661), cleaved caspase 9(7237), cleaved caspase 8(9496), and DR4(42533) from Cell Signaling Technology. DR5(Ab230969) was acquired from Abcam, while TNFR1(60192‐1) and TNFR2(19272‐1) were obtained from Proteintech. The β‐Actin antibody was provided by HuaBio, China.

### 4.6 RNA interfering

The siRNA and RNAiMAX (Invitrogen) were first incubated separately in Opti‐MEM (Invitrogen) for 5 min. Afterward, the solutions were combined and allowed to interact for an additional 15 min. The resulting mixture was added to the cells along with serum‐free medium. The siRNA sequences were custom‐synthesized by Gene‐Pharma. Detailed sequences are provided in the table below.

**Table:** 

siRNA	Sequence (5′−3′)
si‐control	UUCUCCGAACGUGUCACGU
si‐AMPK#1	GGGAACAUGAAUGGUUUAATT
si‐AMPK#2	GGCAUCCUCAUAUAAUUAATT
si‐DR5#1	AAGACCCUUGUGCUCGUUGUC
si‐DR5#2	CAGCCGUAGUCUUGAUUGU

### 4.7 ATP assay

In a six‐well plate, seed 2 × 10^5^ HCC‐LM3 or MHCC97‐H cells. After the cells have adhered to the surface, treat them with silibinin either in a dose‐dependent or time‐dependent manner. And then, remove the culture medium and add lysis buffer according to a ratio of 200 μL per well in the six‐well plate to lyse the cells.

1. Add 100 μL of ATP detection working solution to the testing well or tube. Allow it to sit at room temperature for 3−5 min;
2. To the testing tube, add 20 μL of sample or standard, quickly mix using a pipette (micropipette), allowing at least a 2‐s interval before measuring the relative light units value using a luminometer;
3. Based on the standard curve, calculate the ATP concentration in the samples;
4. Perform a BCA protein concentration assay on the samples to measure ATP production (S0026 ATP Assay Kit; Bytotime).

### 4.8 Glucose assay

2 × 10^5^ cells were seeded in six‐well plates, and then treat them with silibinin in a dose or time‐gradient manner, and collect the cell supernatant as the test samples:

1. Transfer 5 μL of the standard or cell supernatant into 1.5 mL tubes;
2. Add 185 μL of glucose assay reagent, bringing the final volume to 190 μL;
3. After vortex mixing, centrifuge at 5000×*g* for a few seconds to settle the liquid at the bottom of the tubes;
4. Heat in a metal bath at 95°C for 8 min, then cool to 4°C;
5. Transfer 180 μL of the liquid from each tube to a clean 96‐well plate;
6. Measure the absorbance at 630 nm (620–650 nm range can also be used). For optimal results, it is advisable to complete the absorbance measurement within 30 min after the reaction concludes at 95°C;
7. Based on the standard curve, calculate the glucose concentration in the samples. Concurrently, perform a BCA assay to determine the protein concentration, to calculate the glucose content per unit protein. Express the glucose concentration as μg/mg protein (S0201S Glucose Assay Kit with O‐toluidine; Bytotime).

### 4.9 Q300 metabolic profiling

1. Sample preparation: Seed 2 × 10^6^ MHCC97‐H cells in a 10 cm culture dish. Once the cells adhere to the dish, treat them with 200 μM silibinin and DMSO respectively for 48 h, and then collect the samples for analysis;
2. Library construction and sequencing: Send the samples to a Metabo‐Profile Biotechnology (Shanghai) for metabolomic sequencing and library construction;
3. High‐throughput data analysis: Utilize the R programming language to conduct PCA and to generate volcano plots and heatmap visuals.

### 4.10 Establishment of tumor xenograft mouse model

Male balb/c athymic nude mice, aged eight weeks, were purchased from Lingchang Biological Technology, for the tumor formation assay. The animal experimental protocol received approval from the Institutional Animal Care and Use Committee at Longhua Hospital. HCC‐LM3 cells (5 × 10^6^) were suspended in 50 μL PBS and 50 μL matrigel (BD) and injected subcutaneously into the bilateral gluteal regions of the mice. Tumor size was calculated using the formula: length × width.

Eight days postinoculation, the mice underwent fasting with water consumption only. This protocol of fasting has been reported previously. In details, each fasting‐refeeding cycle lasted 48 h, with a 24‐h fasting phase and a 24‐h refeeding phase. After three such cycles, a full feast day was observed.^23^, ^91^, ^92^ For therapeutic interventions, mice were orally gavaged with silibinin at doses of 400 mg/kg/day 5 days a week, for a total of 16 doses. The fasting group and the fasting‐silibinin combination group received either CMC or 400 mg/kg/day silibinin after food withdrawal.

### 4.11 Statistical analysis

Each experiment was replicated thrice, with results presented as mean ± standard error of the mean. Statistical evaluations were conducted using GraphPad Prism 8.3 software. The Student's *t*‐test facilitated intergroup comparisons, where *p < 0.05, **p < 0.01, and ***p < 0.001 indicated statistical significance, and the label n.s. denoted no statistical significance.

## AUTHOR CONTRIBUTION

L. J. and Y. J. designed and supervised the project. B. Y. and Y. J. carried out the experiments, drafted, and finalized manuscript. S. Y., T. X., and L. C. supported with experiment material. All authors read and approved the final manuscript.

## CONFLICT OF INTEREST STATEMENT

The authors declare that there are no competing interests associated with the manuscript.

## ETHICS STATEMENT

The animal study was reviewed and approved by Animal Experimental Ethics Committee of Longhua hospital, Shanghai University of Traditional Chinese Medicine (APPROVAL NUMBER:2020‐N082).

## Data Availability

The original contributions presented in the study are included in the article, and further inquiries can be directed to the corresponding author.

## References

[1] Mcglynn KA , Petrick JL , El‐Serag HB . Epidemiology of hepatocellular carcinoma. Hepatology. 2021;73(1):4‐13. Suppl. 10.1002/hep.31288 PMC7577946 32319693

[2] Bray F , Ferlay J , Soerjomataram I , et al. Global cancer statistics 2018: gLOBOCAN estimates of incidence and mortality worldwide for 36 cancers in 185 countries. CA Cancer J Clin. 2018;68:394‐424. 30207593 10.3322/caac.21492

[3] Petrick JL , Florio AA , Znaor A , et al. International trends in hepatocellular carcinoma incidence, 1978–2012. Int J Cancer. 2020;147:317‐330. 31597196 10.1002/ijc.32723 PMC7470451

[4] Khan AA , Liu ZK , Xu X . Recent advances in immunotherapy for hepatocellular carcinoma. Hepatobiliary Pancreat Dis Int. 2021;20:511‐520. 34344612 10.1016/j.hbpd.2021.06.010

[5] Llovet JM , Zucman‐Rossi J , Pikarsky E , et al. Hepatocellular carcinoma. Nat Rev Dis Primers. 2016;2:16018. 27158749 10.1038/nrdp.2016.18

[6] Zhou J , Zhou F , Wang W , et al. Epidemiological features of NAFLD from 1999 to 2018 in China. Hepatology. 2020;71:1851‐1864. 32012320 10.1002/hep.31150

[7] Colman RJ , Beasley TM , Kemnitz JW , et al. Caloric restriction reduces age‐related and all‐cause mortality in rhesus monkeys. Nat Commun. 2014:5. 10.1038/ncomms4557 PMC3988801 24691430

[8] Fontana L , Partridge L , Longo VD . Extending healthy life span–from yeast to humans. Science. 2010;328:321‐326. 20395504 10.1126/science.1172539 PMC3607354

[9] Fontana L , Partridge L . Promoting health and longevity through diet: from model organisms to humans. Cell. 2015;161:106‐118. 25815989 10.1016/j.cell.2015.02.020 PMC4547605

[10] Longo VD . Mattson M P Fasting: molecular mechanisms and clinical applications. Cell Metab. 2014;19:181‐192. 24440038 10.1016/j.cmet.2013.12.008 PMC3946160

[11] Jordan S , Tung N , Casanova‐Acebes M , et al. Dietary intake regulates the circulating inflammatory monocyte pool. Cell. 2019;178:1102‐1114. e17. 31442403 10.1016/j.cell.2019.07.050 PMC7357241

[12] Di Biase S , Lee C , Brandhorst S , et al. Fasting‐mimicking diet reduces HO‐1 to promote T cell‐mediated tumor cytotoxicity. Cancer Cell. 2016;30:136‐146. 27411588 10.1016/j.ccell.2016.06.005 PMC5388544

[13] Allen BD , Liao CY , Shu J , et al. Hyperadrenocorticism of calorie restriction contributes to its anti‐inflammatory action in mice. Aging Cell. 2019;18:e12944. 30938024 10.1111/acel.12944 PMC6516174

[14] Okada T , Otsubo T , Hagiwara T , et al. Intermittent fasting prompted recovery from dextran sulfate sodium‐induced colitis in mice. J Clin Biochem Nutr. 2017;61:100‐107. 28955126 10.3164/jcbn.17-9 PMC5612824

[15] Meynet O , Ricci JE . Caloric restriction and cancer: molecular mechanisms and clinical implications. Trends Mol Med. 2014;20:419‐427. 24916302 10.1016/j.molmed.2014.05.001

[16] Brandhorst S , Longo V . Fasting and caloric restriction in cancer prevention and treatment. Recent Results Cancer Res. 2016;207:241‐266. 27557543 10.1007/978-3-319-42118-6_12 PMC7476366

[17] Curry NL , Mino‐Kenudson M , Oliver TG , et al. Pten‐null tumors cohabiting the same lung display differential AKT activation and sensitivity to dietary restriction. Cancer Discov. 2013;3:908‐921. 23719831 10.1158/2159-8290.CD-12-0507 PMC3743121

[18] Grasl‐Kraupp B , Bursch W , Ruttkay‐Nedecky B , et al. Food restriction eliminates preneoplastic cells through apoptosis and antagonizes carcinogenesis in rat liver. Proc Natl Acad Sci USA. 1994;91:9995‐9999. 7937932 10.1073/pnas.91.21.9995 PMC44944

[19] Alidadi M , Banach M , Guest PC , et al. The effect of caloric restriction and fasting on cancer. Semin Cancer Biol. 2021;73:30‐44. 32977005 10.1016/j.semcancer.2020.09.010

[20] Clifton KK , Ma CX , Fontana L , Peterson L. Intermittent fasting in the prevention and treatment of cancer. CA Cancer J Clin. 2021;71:527‐546. 34383300 10.3322/caac.21694

[21] Kalaany NY , Sabatini DM . Tumours with PI3K activation are resistant to dietary restriction. Nature. 2009;458:725‐731. 19279572 10.1038/nature07782 PMC2692085

[22] Pietrocola F , Pol J , Vacchelli E , et al. Caloric restriction mimetics enhance anticancer immunosurveillance. Cancer Cell. 2016;30:147‐160. 27411589 10.1016/j.ccell.2016.05.016 PMC5715805

[23] Lee C , Raffaghello L , Brandhorst S , et al. Fasting cycles retard growth of tumors and sensitize a range of cancer cell types to chemotherapy. Sci Transl Med. 2012;4:124ra27. 10.1126/scitranslmed.3003293 PMC3608686 22323820

[24] Krstic J , Reinisch I , Schindlmaier K , et al. Fasting improves therapeutic response in hepatocellular carcinoma through p53‐dependent metabolic synergism. Sci Adv. 2022;8:eabh2635. 35061544 10.1126/sciadv.abh2635 PMC8782451

[25] Lu Z , Xie J , Wu G , et al. Fasting selectively blocks development of acute lymphoblastic leukemia via leptin‐receptor upregulation. Nat Med. 2017;23:79‐90. 27941793 10.1038/nm.4252 PMC6956990

[26] Caffa I , Spagnolo V , Vernieri C , et al. Fasting‐mimicking diet and hormone therapy induce breast cancer regression. Nature. 2020;583:620‐624. 32669709 10.1038/s41586-020-2502-7 PMC7881940

[27] Cavric G , Ilic D , Njers K , Prkacin I , Hamp DB. Native valve endocarditis caused by methicillin‐resistant staphylococcus epidermidis in a patient with advanced liver cirrhosis. Acta Clin Croat. 2015;54:531‐535. 27017731

[28] Levesque S , Le Naour J , Pietrocola F , et al. A synergistic triad of chemotherapy, immune checkpoint inhibitors, and caloric restriction mimetics eradicates tumors in mice. Oncoimmunology. 2019;8:e1657375. 31646107 10.1080/2162402X.2019.1657375 PMC6791453

[29] Madeo F , Carmona‐Gutierrez D , Hofer SJ , Kroemer G . Caloric restriction mimetics against age‐associated disease: targets, mechanisms, and therapeutic potential. Cell Metab. 2019;29:592‐610. 30840912 10.1016/j.cmet.2019.01.018

[30] Nencioni A , Caffa I , Cortellino S , Longo VD . Fasting and cancer: molecular mechanisms and clinical application. Nat Rev Cancer. 2018;18:707‐719. 30327499 10.1038/s41568-018-0061-0 PMC6938162

[31] Hsu CC , Peng D , Cai Z , Lin HK . AMPK signaling and its targeting in cancer progression and treatment. Semin Cancer Biol. 2022;85:52‐68. 33862221 10.1016/j.semcancer.2021.04.006 PMC9768867

[32] Herzig S , Shaw RJ. AMPK: guardian of metabolism and mitochondrial homeostasis. Nat Rev Mol Cell Biol. 2018;19:121‐135. 28974774 10.1038/nrm.2017.95 PMC5780224

[33] Cantó C , Auwerx J . Calorie restriction: is AMPK a key sensor and effector? Physiology. 2011;26:214‐224. 21841070 10.1152/physiol.00010.2011 PMC3627048

[34] Garcia D , Shaw RJ. AMPK: mechanisms of cellular energy sensing and restoration of metabolic balance. Mol Cell. 2017;66:789‐800. 28622524 10.1016/j.molcel.2017.05.032 PMC5553560

[35] Carling D. AMPK signalling in health and disease. Curr Opin Cell Biol. 2017;45:31‐37. 28232179 10.1016/j.ceb.2017.01.005

[36] Zhang CS , Li M , Wang Y , et al. The aldolase inhibitor aldometanib mimics glucose starvation to activate lysosomal AMPK. Nat Metab. 2022;4:1369‐1401. 36217034 10.1038/s42255-022-00640-7 PMC9584815

[37] Martin‐Montalvo A , Mercken EM , Mitchell SJ , et al. Metformin improves healthspan and lifespan in mice. Nat Commun. 2013;4:2192. 23900241 10.1038/ncomms3192 PMC3736576

[38] Barzilai N , Crandall JP , Kritchevsky SB , Espeland M . A metformin as a tool to target aging. Cell Metab. 2016;23:1060‐1065. 27304507 10.1016/j.cmet.2016.05.011 PMC5943638

[39] Pietrocola F , Castoldi F , Markaki M , et al. Aspirin recapitulates features of caloric restriction. Cell Rep. 2018;22:2395‐2407. 29490275 10.1016/j.celrep.2018.02.024 PMC5848858

[40] Pietrocola F , Castoldi F , Maiuri MC , Kroemer G . Aspirin‐another caloric‐restriction mimetic. Autophagy. 2018;14:1162‐1163. 29929449 10.1080/15548627.2018.1454810 PMC6103658

[41] Liu J , Chen D , Liu P , et al. Discovery, synthesis, and structure–activity relationships of 20(S)‐protopanaxadiol (PPD) derivatives as a novel class of AMPKα2β1γ1 activators. Eur J Med Chem. 2014;79:340‐349. 24747289 10.1016/j.ejmech.2014.04.010

[42] Li W , Wang Y , Zhou X , et al. The anti‐tumor efficacy of 20(S)‐protopanaxadiol, an active metabolite of ginseng, according to fasting on hepatocellular carcinoma. J Ginseng Res. 2022;46:167‐174. 35058733 10.1016/j.jgr.2021.06.002 PMC8753519

[43] Price Nathan L , Gomes Ana P , Ling Alvin JY , et al. SIRT1 is required for AMPK activation and the beneficial effects of resveratrol on mitochondrial function. Cell Metab. 2012;15:675‐690. 22560220 10.1016/j.cmet.2012.04.003 PMC3545644

[44] Pearson KJ , Baur JA , Lewis KN , et al. Resveratrol delays age‐related deterioration and mimics transcriptional aspects of dietary restriction without extending life span. Cell Metab. 2008;8:157‐168. 18599363 10.1016/j.cmet.2008.06.011 PMC2538685

[45] Davinelli S , De Stefani D , De Vivo I , Scapagnini G . Polyphenols as caloric restriction mimetics regulating mitochondrial biogenesis and mitophagy. Trends Endocrinol Metab. 2020;31:536‐550. 32521237 10.1016/j.tem.2020.02.011

[46] Jiang Y , Yang X , Dong C , et al. Five‐day water‐only fasting decreased metabolic‐syndrome risk factors and increased anti‐aging biomarkers without toxicity in a clinical trial of normal‐weight individuals. Clin Transl Med. 2021;11:e502. 34459130 10.1002/ctm2.502 PMC8320652

[47] Hanahan D , Weinberg R . A hallmarks of cancer: the next generation. Cell. 2011;144:646‐674. 21376230 10.1016/j.cell.2011.02.013

[48] Pavlova NN , Thompson CB . The emerging hallmarks of cancer metabolism. Cell Metab. 2016;23:27‐47. 26771115 10.1016/j.cmet.2015.12.006 PMC4715268

[49] Warburg OH . The classic: the chemical constitution of respiration ferment. Clin Orthop Relat Res. 2010;468:2833‐2839. 20809165 10.1007/s11999-010-1534-y PMC2947660

[50] Weng ML , Chen WK , Chen XY , et al. Fasting inhibits aerobic glycolysis and proliferation in colorectal cancer via the Fdft1‐mediated AKT/mTOR/HIF1alpha pathway suppression. Nat Commun. 2020;11:1869. 32313017 10.1038/s41467-020-15795-8 PMC7170903

[51] Jiang Y , Tang Z , Zhu X , et al. Non‐invasive omics analysis delineates molecular changes in water‐only fasting and its sex‐discriminating features in metabolic syndrome patients. MedComm. 2023;4:e393. 37929015 10.1002/mco2.393 PMC10622739

[52] Hou S , Zhang T , Li Y , Guo F , Jin X . Glycyrrhizic acid prevents diabetic nephropathy by activating AMPK/SIRT1/PGC‐1alpha signaling in db/db mice. J Diabetes Res. 2017;2017:2865912. 29238727 10.1155/2017/2865912 PMC5697128

[53] Zhang X , Yang H , Yue S , et al. The mTOR inhibition in concurrence with ERK1/2 activation is involved in excessive autophagy induced by glycyrrhizin in hepatocellular carcinoma. Cancer Med. 2017;6:1941‐1951. 28675698 10.1002/cam4.1127 PMC5548872

[54] Khan I , Preeti K , Kumar R , Khatri DK , Singh SB . Activation of SIRT1 by silibinin improved mitochondrial health and alleviated the oxidative damage in experimental diabetic neuropathy and high glucose‐mediated neurotoxicity. Arch Physiol Biochem. 2022:1‐17. 10.1080/13813455.2022.2108454 35943429

[55] Wang C , He C , Lu S , et al. Autophagy activated by silibinin contributes to glioma cell death via induction of oxidative stress‐mediated BNIP3‐dependent nuclear translocation of AIF. Cell Death Disease. 2020;11:630. 32801360 10.1038/s41419-020-02866-3 PMC7429844

[56] Pu Z , Zhang W , Wang M , et al. Schisandrin B attenuates colitis‐associated colorectal cancer through SIRT1 linked SMURF2 signaling. Am J Chin Med. 2021;49:1773‐1789. 34632965 10.1142/S0192415X21500841

[57] Zhu L , Wang Y , Lv W , et al. Schizandrin A can inhibit non‑small cell lung cancer cell proliferation by inducing cell cycle arrest, apoptosis and autophagy. Int J Mol Med. 2021;48:214. 34643254 10.3892/ijmm.2021.5047 PMC8522958

[58] Tang MC , Cheng L , Qiu L , et al. Efficacy of tiopronin in treatment of severe non‐alcoholic fatty liver disease. Eur Rev Med Pharmacol Sci. 2014;18:160‐164. 24488902

[59] Michele Malaguarnera MM . Silybin‐vitamin E‐phospholipids complex reduces liver fibrosis in patients with chronic hepatitis C treated with pegylated interferon α and ribavirin. Am J Transl Res. 2015;7(11):2510‐2518. 26807195 PMC4697727

[60] Loguercio C , Festi D . Silybin and the liver: from basic research to clinical practice. World J Gastroenterol. 2011;17:2288‐2301. 21633595 10.3748/wjg.v17.i18.2288 PMC3098397

[61] Soleimani V , Delghandi PS , Moallem SA , Karimi G . Safety and toxicity of silymarin, the major constituent of milk thistle extract: an updated review. Phytother Res. 2019;33:1627‐1638. 31069872 10.1002/ptr.6361

[62] Brandon‐Warner E , Eheim AL , Foureau DM , et al. Silibinin (Milk Thistle) potentiates ethanol‐dependent hepatocellular carcinoma progression in male mice. Cancer Lett. 2012;326:88‐95. 22863537 10.1016/j.canlet.2012.07.028 PMC3449310

[63] Loguercio C , Andreone P , Brisc C , et al. Silybin combined with phosphatidylcholine and vitamin E in patients with nonalcoholic fatty liver disease: a randomized controlled trial. Free Radic Biol Med. 2012;52:1658‐1665. 22343419 10.1016/j.freeradbiomed.2012.02.008

[64] Xu R , Qiu S , Zhang J , et al. Silibinin Schiff base derivatives counteract CCl(4)‐induced acute liver injury by enhancing anti‐inflammatory and antiapoptotic bioactivities. Drug Des Dev Ther. 2022;16:1441‐1456. 10.2147/DDDT.S356847 PMC9122151 35601675

[65] Fernandes G , Yunis EJ , Good R . A suppression of adenocarcinoma by the immunological consequences of calorie restriction. Nature. 1976;263:504‐507. 10.1038/263504b0 1085916

[66] Jee Sh OH , Sull JW , Yun JE , Ji M , Samet JM . Fasting serum glucose level and cancer risk in korean men and women. JAMA. 2005;293(2):194‐202. 15644546 10.1001/jama.293.2.194

[67] Marinac CR , Nelson SH , Breen CI , et al. Prolonged nightly fasting and breast cancer prognosis. JAMA Oncol. 2016;2:1049‐1055. 27032109 10.1001/jamaoncol.2016.0164 PMC4982776

[68] Bauersfeld SP , Kessler CS , Wischnewsky M , et al. The effects of short‐term fasting on quality of life and tolerance to chemotherapy in patients with breast and ovarian cancer: a randomized cross‐over pilot study. BMC Cancer. 2018;18:476. 29699509 10.1186/s12885-018-4353-2 PMC5921787

[69] Mohamed SY , Emara MH , Hussien HI , ElsadekHM . Changes in portal blood flow and liver functions in cirrhotics during Ramadan fasting in the summer; a pilot study. Gastroenterol Hepatol Bed Bench. 2016;9:180‐188. 27458510 PMC4947132

[70] Elnadry MH , Nigm IA , Abdel Aziz IM , et al. Effect of Ramadan fasting on Muslim patients with chronic liver diseases. J Egypt Soc Parasitol. 2011;41:337‐346. 21980772

[71] Ligorio F , Fuca G , Provenzano L , et al. Exceptional tumour responses to fasting‐mimicking diet combined with standard anticancer therapies: a sub‐analysis of the NCT03340935 trial. Eur J Cancer. 2022;172:300‐310. 35810555 10.1016/j.ejca.2022.05.046

[72] Barzaghi N , Crema F , Gatti G , Pifferi G , Perucca E. Pharmacokinetic studies on IdB 1016, a silybin‐ phosphatidylcholine complex, in healthy human subjects. Eur J Drug Metab Pharmacokinet. 1990;15:333‐338. 2088770 10.1007/BF03190223

[73] Weyhenmeyer R , Mascher H , Birkmayer J . Study on dose‐linearity of the pharmacokinetics of silibinin diastereomers using a new stereospecific assay. Int J Clin Pharmacol Ther Toxicol. 1992;30:134‐138. 1572758

[74] Wah Kheong C , Nik Mustapha NR , Mahadeva S . A randomized trial of silymarin for the treatment of nonalcoholic steatohepatitis. Clin Gastroenterol Hepatol. 2017;15:1940‐1949. e8. 28419855 10.1016/j.cgh.2017.04.016

[75] Siegel AB , Stebbing J . Milk thistle: early seeds of potential. Lancet Oncol. 2013;14:929‐930. 23993379 10.1016/S1470-2045(13)70414-5 PMC4116427

[76] Reagan‐Shaw S , Nihal M , Ahmad N. Dose translation from animal to human studies revisited. FASEB J. 2008;22:659‐661. 17942826 10.1096/fj.07-9574LSF

[77] Li L , Zeng J , Gao Y , He D. Targeting silibinin in the antiproliferative pathway. Expert Opin Investig Drugs. 2010;19:243‐255. 10.1517/13543780903533631 20047507

[78] Tuli HS , Mittal S , Aggarwal D , et al. Path of Silibinin from diet to medicine: a dietary polyphenolic flavonoid having potential anti‐cancer therapeutic significance. Semin Cancer Biol. 2021;73:196‐218. 33130037 10.1016/j.semcancer.2020.09.014

[79] Kamran MZ , Patil P , Gude RP . Role of STAT3 in cancer metastasis and translational advances. Biomed Res Int. 2013;2013:421821. 24199193 10.1155/2013/421821 PMC3807846

[80] Si L , Fu J , Liu W , et al. Silibinin inhibits migration and invasion of breast cancer MDA‐MB‐231 cells through induction of mitochondrial fusion. Mol Cell Biochem. 2020;463:189‐201. 31612353 10.1007/s11010-019-03640-6

[81] Riebold M , Kozany C , Freiburger L , et al. A C‐terminal HSP90 inhibitor restores glucocorticoid sensitivity and relieves a mouse allograft model of Cushing disease. Nat Med. 2015;21:276‐280. 25665180 10.1038/nm.3776

[82] Verdura S , Cuyas E , Llorach‐Pares L , et al. Silibinin is a direct inhibitor of STAT3. Food Chem Toxicol. 2018;116:161‐172. 29660364 10.1016/j.fct.2018.04.028

[83] Zhao P , Sun X , Chaggan C , et al. An AMPK‐caspase‐6 axis controls liver damage in nonalcoholic steatohepatitis. Science. 2020;367:652‐660. 32029622 10.1126/science.aay0542 PMC8012106

[84] Wang C , He H , Liu G , et al. DT‐13 induced apoptosis and promoted differentiation of acute myeloid leukemia cells by activating AMPK‐KLF2 pathway. Pharmacol Res. 2020;158:104864. 32416217 10.1016/j.phrs.2020.104864

[85] Madeo F , Pietrocola F , Eisenberg T , Kroemer G. Caloric restriction mimetics: towards a molecular definition. Nat Rev Drug Discov. 2014;13:727‐740. 25212602 10.1038/nrd4391

[86] Wei M , Brandhorst S , Shelehchi M , et al. Fasting‐mimicking diet and markers/risk factors for aging, diabetes, cancer, and cardiovascular disease. Sci Transl Med. 2017;9(377):eaai8700. 28202779 10.1126/scitranslmed.aai8700 PMC6816332

[87] Ingram DK , Zhu M , Mamczarz J , et al. Calorie restriction mimetics: an emerging research field. Aging Cell. 2006;5:97‐108. 16626389 10.1111/j.1474-9726.2006.00202.x

[88] Ren G , Ma Y , Wang X , Zheng Z , Li G . Aspirin blocks AMPK/SIRT3‐mediated glycolysis to inhibit NSCLC cell proliferation. Eur J Pharmacol. 2022;932:175208. 35981603 10.1016/j.ejphar.2022.175208

[89] Raina K , Agarwal C , Wadhwa R , Serkova NJ , Agarwal R . Energy deprivation by silibinin in colorectal cancer cells: a double‐edged sword targeting both apoptotic and autophagic machineries. Autophagy. 2013;9:697‐713. 23445752 10.4161/auto.23960 PMC3669180

[90] Garcia‐Maceira P , Mateo J . Silibinin inhibits hypoxia‐inducible factor‐1alpha and mTOR/p70S6K/4E‐BP1 signalling pathway in human cervical and hepatoma cancer cells: implications for anticancer therapy. Oncogene. 2009;28:313‐324. 18978810 10.1038/onc.2008.398

[91] Chen X , Lin X , Li M . Comprehensive modulation of tumor progression and regression with periodic fasting and refeeding circles via boosting IGFBP‐3 loops and NK responses. Endocrinology. 2012;153:4622‐4632. 22903617 10.1210/en.2011-2101

[92] Sun P , Wang H , He Z , et al. Fasting inhibits colorectal cancer growth by reducing M2 polarization of tumor‐associated macrophages. Oncotarget. 2017;8:74649‐74660. 29088814 10.18632/oncotarget.20301 PMC5650369

